# Supplementary material for: SARS-CoV-2 exposure, symptoms and seroprevalence in healthcare workers in Sweden
Source: Nat Commun. 2020 Oct 8;11:5064. doi: 10.1038/s41467-020-18848-0 (PMC7544689; doi:10.1038/s41467-020-18848-0)
Supplement: Supplementary file 1 — Supplementary information [file 41467_2020_18848_MOESM1_ESM.pdf]

Supplementary information

Supplementary table 1. Sensitivity and specificity for each antigen and the combined results requiring reactivity for at least two out of the three antigens. Confidence intervals were computed using the binomial test. Source data are available as Source Data file.

	**Positive controls (n=243)**				**Negative controls (n=442)**			
	Not reactive	Reactive	Sensitivity (%)	95% CI	Not reactive	Reactive	Specificity (%)	95% CI
Nucleocapsid protein	8	235	96.7	93.6-98.6	435	7	98.4	96.8-99.4
Spike S1 domain	2	241	99.2	97.1-99.9	438	4	99.1	97.7-99.8
Spike trimer	1	242	99.6	97.7-100	437	5	98.9	97.4-99.6
**Combined result (2/3 positive antigens)**	2	241	99.2	97.1-99.9	441	1	99.8	98.7-100

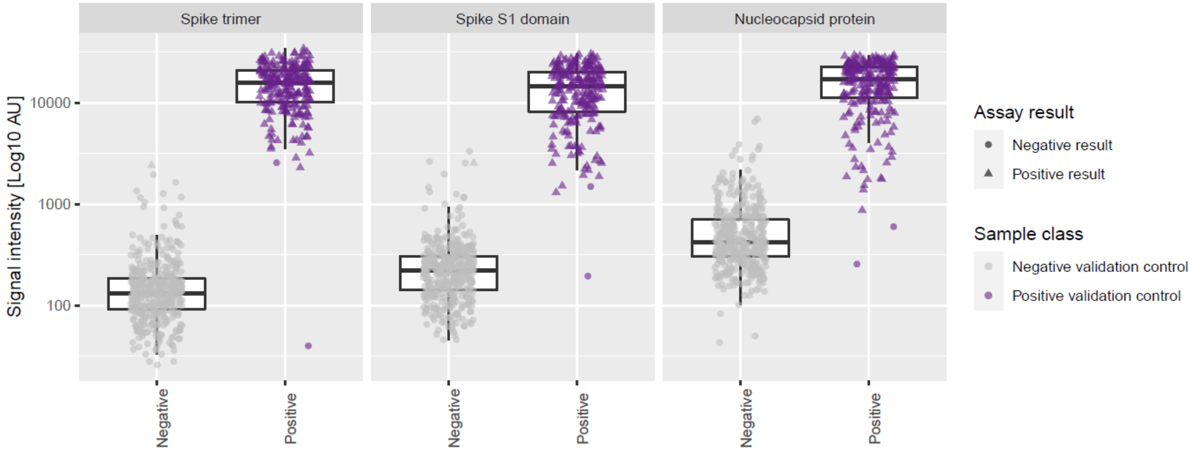

Supplementary figure 1.

Results for the 243 positive controls and the 442 negative controls for each of the three antigens. Boxes encompass the first to third quartiles, with the horizontal bar denoting the median, and the whiskers denoting 1.5 times the interquartile range above and below the first and third quartiles, respectively. Source data are available as Source Data file.
