# Supplementary material for: SARS-CoV-2 exposure, symptoms and seroprevalence in healthcare workers in Sweden
Source: Nat Commun. 2020 Oct 8;11:5064. doi: 10.1038/s41467-020-18848-0 (PMC7544689; doi:10.1038/s41467-020-18848-0)
Supplement: Supplementary file 3 — reporting-summary [file 41467_2020_18848_MOESM3_ESM.pdf]

nature research

Corresponding author(s): Charlotte Thålin

Last updated by author(s): Sep 9, 2020

Reporting Summary

Nature Research wishes to improve the reproducibility of the work that we publish. This form provides structure for consistency and transparency in reporting. For further information on Nature Research policies, see our Editorial Policies and the Editorial Policy Checklist.

Statistics

For all statistical analyses, confirm that the following items are present in the figure legend, table legend, main text, or Methods section.

n/a	Confirmed	
☐	☒	The exact sample size (*n*) for each experimental group/condition, given as a discrete number and unit of measurement
☒	☐	A statement on whether measurements were taken from distinct samples or whether the same sample was measured repeatedly
☐	☒	The statistical test(s) used AND whether they are one- or two-sided<br>*Only common tests should be described solely by name; describe more complex techniques in the Methods section.*
☐	☒	A description of all covariates tested
☐	☒	A description of any assumptions or corrections, such as tests of normality and adjustment for multiple comparisons
☐	☒	A full description of the statistical parameters including central tendency (e.g. means) or other basic estimates (e.g. regression coefficient) AND variation (e.g. standard deviation) or associated estimates of uncertainty (e.g. confidence intervals)
☒	☐	For null hypothesis testing, the test statistic (e.g. *F*, *t*, *r*) with confidence intervals, effect sizes, degrees of freedom and *P* value noted<br>*Give P values as exact values whenever suitable.*
☒	☐	For Bayesian analysis, information on the choice of priors and Markov chain Monte Carlo settings
☒	☐	For hierarchical and complex designs, identification of the appropriate level for tests and full reporting of outcomes
☒	☐	Estimates of effect sizes (e.g. Cohen's *d*, Pearson's *r*), indicating how they were calculated

Our web collection on statistics for biologists contains articles on many of the points above.

Software and code

Policy information about availability of computer code

Data collection	No commercial, open source or custom code was used to collect the data in this study.
Data analysis	Statistical analyses and visualizations were performed in R 3.6.3, using packages tidyverse 1.3.0, lubridate 1.7.4, rlang 0.4.6, pander 0.6.3, knitr 1.28, and UpSetR 1.4.0 (RStudio Team 2019, Boston, USA).

For manuscripts utilizing custom algorithms or software that are central to the research but not yet described in published literature, software must be made available to editors and reviewers. We strongly encourage code deposition in a community repository (e.g. GitHub). See the Nature Research guidelines for submitting code & software for further information.

Data

Policy information about availability of data

All manuscripts must include a data availability statement. This statement should provide the following information, where applicable:

- Accession codes, unique identifiers, or web links for publicly available datasets
- A list of figures that have associated raw data
- A description of any restrictions on data availability

The anonymized datasets generated during and/or analyzed during the current study are available from the corresponding author on reasonable request. Source data are provided with this paper.

Field-specific reporting

Please select the one below that is the best fit for your research. If you are not sure, read the appropriate sections before making your selection.

☒ Life sciences ☐ Behavioural & social sciences ☐ Ecological, evolutionary & environmental sciences

For a reference copy of the document with all sections, see nature.com/documents/nr-reporting-summary-flat.pdf

Life sciences study design

All studies must disclose on these points even when the disclosure is negative.

Sample size	No sample size calculation was performed. The sample size was based on assumed number of employees willing and able to particiapate in the study. Sample size was a priori set to 2000, but when we reached 2149 participants there were still employees willing to participate. Due to logistics and avalilable funding at the time, study inclusion was stopped at 2149 participants.
Data exclusions	There were no exclusion criteria.
Replication	The generated data was validated through extensive analysis of the reproducibility in and between every assay where all positive controls and negative controls were compared between each assay.<br>Four positive controls were re-run on every assay-plate and had a mean inter-assay coefficient of variation of 10.1% (8.0-13.3%) based on absolute intensity levels.
Randomization	The study is an observational study and there was no intervention. We therefore did not implement a randomization.
Blinding	all sample providers had an unknown serology status for SARS-CoV-2 and thereby randomized and the degree of symptom and any other individual data was blinded at the analysis until all the samples were analysed.

Reporting for specific materials, systems and methods

We require information from authors about some types of materials, experimental systems and methods used in many studies. Here, indicate whether each material, system or method listed is relevant to your study. If you are not sure if a list item applies to your research, read the appropriate section before selecting a response.

Materials & experimental systems

n/a	Involved in the study	
☐	☒	Antibodies
☒	☐	Eukaryotic cell lines
☒	☐	Palaeontology and archaeology
☒	☐	Animals and other organisms
☐	☒	Human research participants
☐	☒	Clinical data
☒	☐	Dual use research of concern

Methods

n/a	Involved in the study	
☒	☐	ChIP-seq
☒	☐	Flow cytometry
☒	☐	MRI-based neuroimaging

Antibodies

Antibodies used	Serum IgG bound to antigen coated beads was detected by F(ab')2-Goat anti-Human IgG Fc Secondary Antibody, PEfluorescent anti-hIgG (Invitrogen, H10104. Validation procedure is described at www.thermofisher.com/se/en/home/life-science/antibodies/invitrogen-antibody-validation.html) and recorded as relative fluorescence intensity (AU).
Validation	*Describe the validation of each primary antibody for the species and application, noting any validation statements on the manufacturer's website, relevant citations, antibody profiles in online databases, or data provided in the manuscript.*

Human research participants

Policy information about studies involving human research participants

Population characteristics	A total of 2149 HCW were included in the study. The majority of study participants were women (85%) and the mean age was 44 (SD 12) years. We did not collect additional data such as comorbidities and ongoing medications.
Recruitment	All employees at Danderyd Hospital (n=4375) were invited by e-mail and through information on the hospital intranet to participate in the study. Consecutive study inclusion took place between 15th of April to 8th of May 2020. Participants were eligible to participate in the study irrespective of whether they had had symptoms since the covid-19 outbreak onset or not. There were no exclusion criteria. Study participation was voluntary.
Ethics oversight	The study protocol was approved by the Swedish Regional Ethical Review Board (dnr 2020-01653). There is no institutet ethical committee.

Note that full information on the approval of the study protocol must also be provided in the manuscript.

Clinical data

Policy information about clinical studies

All manuscripts should comply with the ICMJEguidelines for publication of clinical research and a completedCONSORT checklist must be included with all submissions.

Clinical trial registration	Not registered on ClinicalTrials.gov
Study protocol	Full trial protocol can be accessed on demand
Data collection	Consecutive study inclusion took place between 15th of April to 8th of May 2020. Samples were analysed for serological data at SciLifeLab, KTH, Stockholm.
Outcomes	Primary outcome was IgG antibodies against SARS-CoV-2. This was related to prior symptoms and to type of work exposure.
